# Comparison of 6 cycles with 4 cycles of chemotherapy and atezolizumab in the first-line treatment of ES-SCLC: a retrospective multicenter analysis

**DOI:** 10.1093/oncolo/oyag092

**Published:** 2026-03-16

**Authors:** Halil Göksel Güzel, Derya Kıvrak Salim, Saadettin Kılıçkap, Yasemin Kemal, Cengiz Akosman, Çağlar Köseoğlu, Alper Coşkun, Seval Orman, Safa Can Efil, Onur Baş, Esra Zeynelgil, Halil Çelik, Aytaç Terzi, Tuba Karaçelik, Nuri Karadurmuş, Erdem Çubukçu, Tuğba Başoğlu Tüylü, Burak Bilgin, Mustafa Erman, Serdar Karakaya, Kadir Eser, Elif Atağ, Melek Karakurt Eryılmaz, Banu Öztürk

**Affiliations:** Department of Medical Oncology, Antalya Training and Research Hospital, Antalya, 07000, Turkey; Department of Medical Oncology, Antalya Training and Research Hospital, Antalya, 07000, Turkey; Department of Medical Oncology, Ankara Liv Hospital, Ankara, 06000, Turkey; Department of Medical Oncology, Samsun Medical Park Hospital, Samsun, 55000, Turkey; Department of Medical Oncology, Ordu Medical Park Hospital, Ordu, 52000, Turkey; Department of Medical Oncology, Gülhane Training and Research Hospital, Ankara, 06000, Turkey; Department of Medical Oncology, Bursa Uludağ University Hospital, Bursa, 16000, Turkey; Department of Medical Oncology, Kartal Dr. Lütfi Kırdar City Hospital, İstanbul, 34000, Turkey; Department of Medical Oncology, Ankara Bilkent City Hospital, Ankara, 06000, Turkey; Department of Medical Oncology, Hacettepe University Hospital, Ankara, 06000, Turkey; Department of Medical Oncology, Atatürk Senatoryum Training and Research Hospital, Ankara, 06000, Turkey; Department of Medical Oncology, Mersin University Hospital, Mersin, 33000, Turkey; Department of Medical Oncology, Dokuz Eylül University Hospital, İzmir, 35000, Turkey; Department of Medical Oncology, Necmettin Erbakan University Hospital, Konya, 42000, Turkey; Department of Medical Oncology, Gülhane Training and Research Hospital, Ankara, 06000, Turkey; Department of Medical Oncology, Bursa Uludağ University Hospital, Bursa, 16000, Turkey; Department of Medical Oncology, Kartal Dr. Lütfi Kırdar City Hospital, İstanbul, 34000, Turkey; Department of Medical Oncology, Ankara Bilkent City Hospital, Ankara, 06000, Turkey; Department of Medical Oncology, Hacettepe University Hospital, Ankara, 06000, Turkey; Department of Medical Oncology, Atatürk Senatoryum Training and Research Hospital, Ankara, 06000, Turkey; Department of Medical Oncology, Mersin University Hospital, Mersin, 33000, Turkey; Department of Medical Oncology, Dokuz Eylül University Hospital, İzmir, 35000, Turkey; Department of Medical Oncology, Necmettin Erbakan University Hospital, Konya, 42000, Turkey; Department of Medical Oncology, Antalya Training and Research Hospital, Antalya, 07000, Turkey

**Keywords:** small-cell lung cancer, atezolizumab, chemoimmunotherapy

## Abstract

**Aim:**

IMpower133 was designed with 4 cycles of chemoimmunotherapy with atezolizumab, unlike many other studies permitted 6-cycle induction. This study aimed to compare the efficacy and safety of a 4-cycle regimen with an extended 6-cycle regimen of carboplatin, etoposide, and atezolizumab in the first-line treatment of extensive-stage small-cell lung cancer (ES-SCLC).

**Methods:**

This retrospective multicenter study was conducted across 13 oncology centers in Turkey. A total of 181 patients with ES-SCLC who received first-line treatment with chemotherapy plus atezolizumab were analyzed and grouped into those receiving 4 cycles (*n* = 101) and 6 cycles (*n* = 80) of treatment.

**Results:**

The median follow-up time was 20.9 months. The objective response rates were similar between the 4- and 6-cycle groups (79.2% vs 78.8%, *P* = .940). The disease control rates were also comparable (84.2% vs 86.3%, *P* = .940). The median progression-free survival (PFS) was 6.3 months in the 4-cycle and 8.1 months in the 6-cycle group (*P* = .357). The median overall survival (OS) was 16.6 months for the 4-cycle and 13.6 months for the 6-cycle group (*P* = .583). Intracranial progression was higher in the 4-cycle group (50.0% vs 25.5%, *P* = .007). Immune-related adverse events were similar between the groups.

**Conclusion:**

This is one of the largest real-world datasets comparing the efficacy and safety of 4 versus 6 cycles of induction chemoimmunotherapy. Although there were no significant improvements in PFS or OS, the 6-cycle regimen was associated with a lower observed rate of intracranial progression, predominantly among patients without baseline brain metastases. Further studies are needed to identify this subject in future.

Implications for Practice Extending the carboplatin, etoposide, and atezolizumab induction to 6 cycles did not improve either PFS or OS comparing to standard 4-cycle regimen. However, it may be considered in patients who do not have baseline brain metastasis, since the intracranial progression rate is significantly lower in the 6-cycle group of our study and this effect of prolonged treatment was observed predominantly in patients without baseline brain metastasis. The immune-related adverse events were similar across the 6-cycle and the 4-cycle groups.

## Introduction

Small-cell lung cancer (SCLC) accounts for 15% of all lung cancers and is strongly associated with smoking.^1^ In clinical practice, SCLC is classified as limited-stage small-cell lung cancer (LS-SCLC) and extensive-stage small-cell lung cancer (ES-SCLC). LS-SCLC is a disease confined to one hemithorax and can be encompassed in the radiotherapy field with the exclusion of pleural, pericardial, and extrathoracic metastases. ES-SCLC is any disease burden that cannot be characterized as LS-SCLC.^2^ The prognosis of ES-SCLC was poor before the use of immune checkpoint inhibitors (ICIs), with a 5-year survival rate of <5%.^3^ For almost 3 decades, platinum-based chemotherapy has been the standard of treatment. All efforts to improve survival, including the extension of chemotherapy from 4 cycles to 6 cycles, could only lead to numerical improvements rather than creating a significant difference.^4^^,^^5^ After starting the use of ICIs in first-line treatment in combination with chemotherapy, the 5-year overall survival (OS) rates exceeded 10%.^3^

In 2018, atezolizumab was the first ICI to show both progression-free survival (PFS) and OS benefits over placebo when added to carboplatin plus etoposide (Carbo/Etop) in the IMpower133 phase-3 study. Atezolizumab in combination with Carbo/Etop was administered for 4 cycles (Q3W) and atezolizumab maintenance was continued in patients without progression.^6^ The clinical benefit of atezolizumab was independent of programmed death ligand-1 (PD-L1) status, tumor mutation burden status, or other biomarkers.^7^ Most subsequent first-line chemoimmunotherapy trials adopted a 4-cycle induction design similar to IMpower133, although some permitted up to 6 cycles.^8^^,^^9^ Each of these ICIs provided a significant improvement in PFS and OS compared with placebo plus chemotherapy.^10^

This study aimed to evaluate the differences in efficacy and safety between the standard 4-cycle administration of atezolizumab plus carbo/etop combined induction regimen and its extension to a 6-cycle administration in the first-line treatment of patients diagnosed with ES-SCLC.

## Methods

### Study population

We conducted a retrospective study and screened data from 13 medical oncology centers in various regions of Turkey. A total of 194 patients with ES-SCLC (*n* = 194) treated with carbo/etop plus atezolizumab between January 2019 and November 2024 were identified from medical records. Prior LS-SCLC therapy was allowed if it was completed ≥3 months before the ES-SCLC diagnosis. Patients who received less than 4 cycles (*n* = 5), 5 cycles (*n* = 6), or more than 6 cycles (*n* = 2) of carbo/etop plus atezolizumab combination therapy were excluded from the study. A total of 181 patients (*n* = 181) were included in the final analysis. The patients were grouped into the 4-cycle group (*n* = 101) received standard 4 cycles of carbo/etop and atezolizumab, and the 6-cycle group (*n* = 80) received extended 6 cycles of induction chemoimmunotherapy. Given the absence of strict recommendations regarding prophylactic cranial irradiation (PCI) in extensive-stage small-cell lung cancer, decisions concerning PCI were individualized, based on real-world physician–patient collaboration.

In this study, both 4- and 6-cycle induction approaches were used throughout the study period, and the treatment duration was not restricted to a specific calendar interval. The number of induction cycles was determined according to the treating physician’s decision, treatment tolerance, disease control, and prevailing clinical practice patterns at the time of treatment. In the 4-cycle group, carbo/etop discontinuation after 4 cycles most commonly reflected the completion of planned induction therapy in accordance with guideline-based practice, while disease progression was a reason for discontinuation in a minority of cases. Importantly, patients who experienced early disease progression or treatment intolerance were inherently less likely to receive extended induction therapy. Consequently, the 6-cycle cohort may have been enriched for patients with more favorable disease biology and treatment tolerance, introducing potential selection bias. This limitation is inherent to retrospective comparisons based on treatment exposure and should be considered when interpreting the efficacy and survival outcomes.

This study complied with the tenets of the Declaration of Helsinki. Ethical committee approval was obtained from the Antalya Training and Research Hospital Ethical Committee (Approval No: 3/15, 2024-44).

### Data collection

Data entry included patients’ demographic information, Eastern Cooperative Oncology Study Group performance status (ECOG PS), body mass index, Charlson comorbidity index, presence of any oncological emergencies at the time of ES-SCLC diagnosis, metastasis sites, radiotherapy history, tumor response evaluations, post-progression treatment selections, and immune-related adverse reactions (irAEs) and the type and severity of the irAEs were recorded in the patient follow-up files by the treating physician at each center in accordance with the Common Terminology Criteria for Adverse Reactions version 5 (CTCAE v5). irAEs were identified through a retrospective review of the medical records and physician documentation. Adverse events were classified according to the CTCAE v5 when sufficient clinical details were available; however, given the retrospective design, systematic prospective toxicity monitoring was not feasible. Accordingly, irAE reporting should be interpreted as a clinically documented event, rather than a protocol-driven toxicity assessment. In the cohort receiving 4 cycles of chemoimmunotherapy, response assessment was performed following the completion of the fourth cycle. For patients treated with 6 cycles, the results of imaging studies conducted after the sixth cycle were recorded. Imaging results obtained during earlier interim cycles, if available, were not included in this study. Tumor response categories were assigned using the response evaluation criteria in solid tumors version 1.1 (RECIST v1.1) terminology based on contemporaneous clinical radiology reports.^11^ Tumor response was assessed based on contemporaneous clinical radiology reports generated during routine care. A formal central review or re-measurement of the imaging studies was not conducted. The RECIST v1.1 terminology was used descriptively to categorize response patterns, rather than to replicate the rigor of prospective trial-based RECIST assessments. The Charlson comorbidity index was dichotomized with a cutoff point of 9.^12^ The disease stage was specified as limited or extensive depending on the tumor spread out of the originated hemithorax.

### Statistical analyses

IBM SPSS v24.0 was used for the statistical analyses. Descriptive statistics were presented as percentages and frequency distributions. Continuous variables are reported as medians (ranges). Comparative analysis of independent categorical variables was performed using the Chi-Square or Fischer’s Exact test. The objective response rate was defined as the percentage of patients who achieved complete response (CR) or partial response (PR). The disease control rate was defined as the proportion of patients who achieved CR, PR, or stable disease (SD). PFS was calculated from the initiation of first-line treatment until disease progression or death. Given the retrospective nature of the study, radiologic assessments were performed according to routine clinical practice at each participating center, and the imaging intervals were not pre-specified or uniform across patients. Therefore, PFS should be interpreted as a time-to-event outcome reflecting real-world clinical decision-making, rather than a strictly standardized endpoint comparable to that of prospective clinical trials. OS was calculated as the duration between the initiation of treatment and death. Survival analyses were performed using Kaplan–Meier analysis. Comparative analyses of survival between the 4- and 6-cycle groups were performed using log-rank tests. Univariate and multivariate analyses were performed using the Cox proportional hazards regression test. Hazard ratios (HR) were calculated for each variable with 95% CI. Variables with univariate *P* < .30 were entered into the multivariate Cox model. A *P*-value <.05 was considered statistically significant.

## Results

### General characteristics and comparison of clinical features between the study groups

A total of 181 patients treated with carbo/etop plus atezolizumab for SCLC from 13 centers were analyzed (*n* = 181). The median follow-up duration was 20.9 months; 95% CI (16.7-25.1). The median age was 62.4 years (38.0-88.4). Eighty patients received 6 cycles of carbo/etop plus atezolizumab (6-cycle group, *n* = 80), and 101 received 4 cycles (4-cycle group, *n* = 101). There were 20 females (19.8%) in the 4-cycle group and 17 females (21.3%) in the 6-cycle group (*P* = .810). Charlson comorbidity index was ≥9 in 68 patients (67.3%) in the 4-cycle group and 55 patients (68.8%) in the 6-cycle group (*P* = .839). Seventy-seven patients (76.2%) in the 4-cycle group and 65 patients (81.3%) in the 6-cycle group were ECOG PS 0 or 1 (*P* = .415). Considering the patients with ECOG PS ≥2, one patient in each group had ECOG PS 3, and none of the patients had ECOG PS 4. Eight patients (7.9%) in the 4-cycle group and 7 patients (8.8%) in the 6-cycle group presented with an oncological emergency at the time of ES-SCLC diagnosis (*P* = .841). The number of metastatic sites was ≥3 in 48.5% and 53.8% of patients in the 4- and 6-cycle groups, respectively (*P* = .484). Brain and liver metastasis rates were comparable (Table 1).

**Table 1:** Comparison of clinical features between the study groups.

	Four-cycle group (*n* = 101)	Six-cycle group (*n* = 80)	*P* value
**Age, *n* (%)**			.514
** <65 years**	57 (56.4)	49 (61.3)	
** ≥65 years**	44 (41.9)	31 (33.1)	
**Sex, *n* (%)**			.810
** Female**	20 (19.8)	17 (21.3)	
** Male**	81 (80.2)	63 (78.8)	
**ECOG PS, *n* (%)**			.415
** 0-1**	77 (76.2)	65 (81.3)	
** ≥2**	24 (21.8)	15 (17.2)	
**Smoking status, *n* (%)**			.522
** Active smoker**	62 (61.4)	53 (66.3)	
** Former smoker**	38 (37.6)	25 (31.3)	
** Never smoker**	1 (1.0)	2 (2.5)	
**Charlson Comorbidity Index, *n* (%)**			.839
** <9**	33 (32.7)	25 (31.3)	
** ≥9**	68 (67.3)	55 (68.8)	
**Body mass index^a^ (kg/m^2^), *n* (%)**			.683
** <25**	55 (55.6)	42 (52.5)	
** ≥25**	44 (44.4)	38 (47.5)	
**Stage at initial diagnosis**			
** Limited stage**	8 (7.9)	2 (2.5)	.113
** Extensive stage**	93 (92.1)	78 (97.5)	
**Oncological emergencies at presentation, *n* (%)**			.841
** VCSS**	4	5	
** Increased intracranial pressure**	1	1	
** Hypercalcemia**	1	1	
** Other**	2	1	
**Metastatic sites, *n* (%)**			
** Brain metastasis**	18 (17.8)	20 (25.0)	.239
** Liver metastasis**	35 (34.7)	39 (48.8)	.055
**Number of metastatic sites**			.484
** <3**	52 (51.5)	37 (46.3)	
** ≥3**	49 (48.5)	43 (53.8)	
**Radiotherapy details, *n* (%)**			
** PCI**	19 (18.8)	16 (20.0)	.841
** Palliative cranial radiotherapy**	15 (14.9)	13 (16.3)	.796
** Palliative thoracic radiotherapy**	7 (6.9)	9 (11.3)	.309

Abbreviations: ECOG PS, Eastern Cooperative Oncology Study Group performance status; PCI, prophylactic cranial irradiation; VCSS, superior vena cava syndrome.

^a^Body mass index was not available for 2 patients in the 4-cycle group.

#### Comparison of treatment efficacy, survival, and immunotherapy-related adverse events between the study groups

The median follow-up times in the 4-and 6-cycle groups were 22.0 months; 95% CI (19.0-24.9) and 20.3 months; 95% CI (4.6-36.1), respectively. The median atezolizumab maintenance was 3 (0-66) vs 2 (0-28) cycles in the 4- vs 6-cycle groups. The 4- and 6-cycle groups had similar objective response rates (79.2% and 78.8%, respectively; *P* = .940) and disease control rates (84.2% and 86.3%, respectively; *P* = .695).

**Figure 1:**
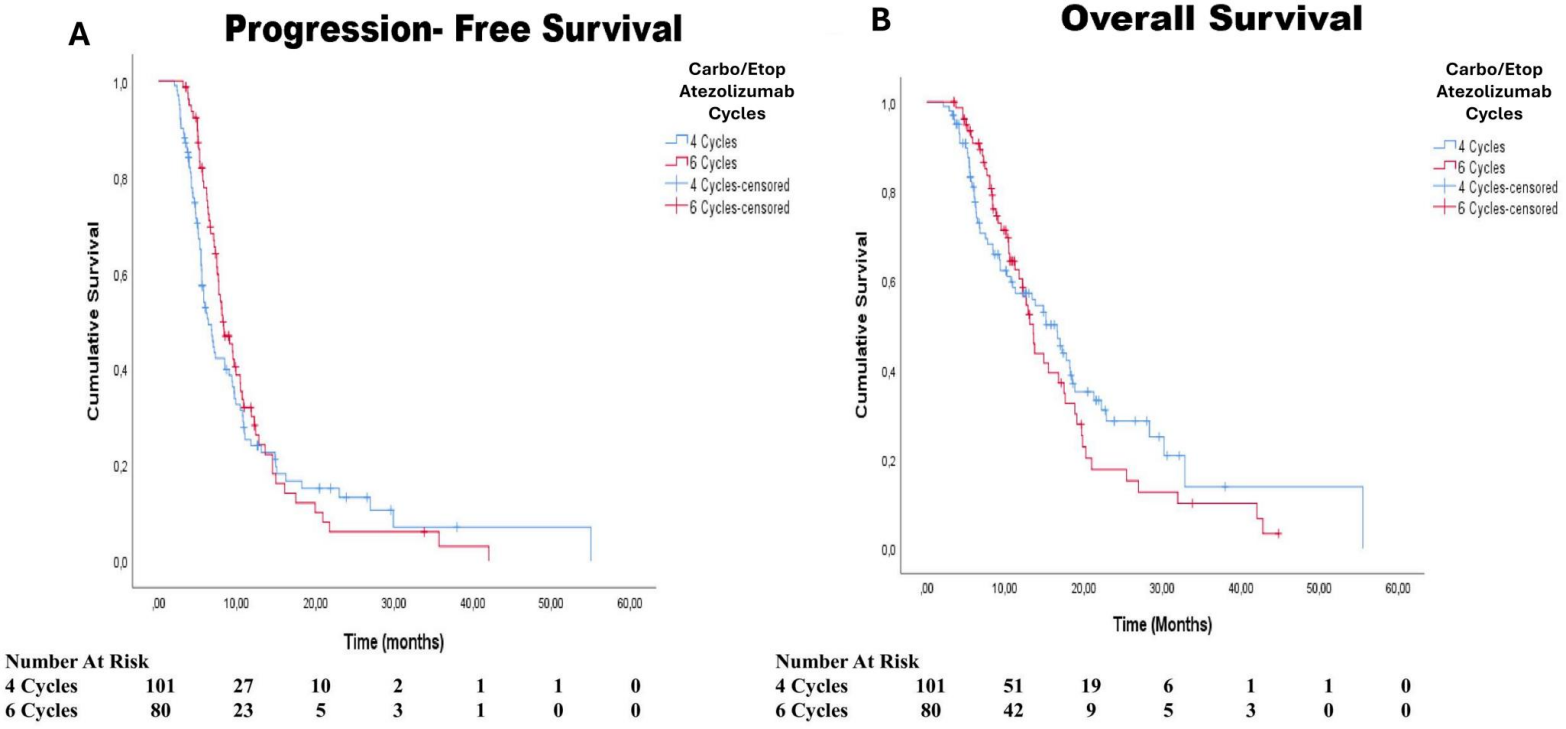
Survival outcomes according to chemotherapy cycle number. (A) Progression-free survival (PFS) according to the duration of induction chemotherapy. The median PFS was 6.3 months (95% CI, 5.2-7.4) in the 4-cycle group and 8.1 months (95% CI, 6.4-9.8) in the 6-cycle group, with no statistically significant difference between the groups (log-rank, *P* = .357). (B) Overall survival (OS) according to the induction chemotherapy duration. The median OS was 16.6 months (95% CI, 13.1-20.0) in the 4-cycle group and 13.6 months (95% CI, 12.1-15.1) in the 6-cycle group (log-rank, *P* = .583).

Seventy-nine patients (78.2%) in the 4-cycle group and 63 patients (78.8%) in the 6-cycle group had disease progression. The median PFS was similar between the 4- and 6-cycle groups (6.3 months; 95% CI: 5.2-7.4 and 8.1 months; 95% CI: 6.4-9.8, respectively; log-rank, *P* = .357) (Figure 1A). The median OS was also similar between the 4- and 6-cycle groups (16.6 months 95% CI: 13.1-20.0 and 13.6 months; 95% CI: 12.1-15.1, respectively; log-rank, *P* = .583) (Figure 1B).

The progression sites were not available for 11 of the 79 patients (13.9%) in the 4-cycle group and for 12 of the 63 patients (19.0%) in the 6-cycle group. Those with missing data were not included in the following progression site comparison analysis but were included in the survival and adverse event analyses. The intracranial progression rate was significantly higher in the 4-cycle group (34 patients, 50.0%) than in the 6-cycle group (13 patients, 25.5%), (*P* = .007). The progression with a liver metastasis rate was significantly lower in the 4-cycle group (27.9% and 47.1%, respectively; *P* = .032) (Table 2). Thirty-five of the 47 intracranial progression events (74.5%) were in patients who did not have brain metastasis previously. Of the 34 intracranial progression events in the 4-cycle group 28(82.4%) were observed in patients without baseline brain metastasis, while it was 7 events (53.8%) for the 6-cycle group, (*P* = .004). The liver progression event ratio in patients without baseline liver metastasis was 27.9% (12 of the 43 patients). The proportion of liver progression events in patients without baseline liver metastasis was 3 (7.3%) in the 4-cycle group and 9 (37.5%) in the 6-cycle group (*P* = .002).

**Table 2:** Progression sites comparison between the study groups.

	Four-cycle group (*n* = 101)	Six-cycle group (*n* = 80)	*P* value
**Any progression, *n* (%)**	79 (78.2)	63 (78.8)	
**Intracranial progression^a^, *n* (%)**	34 (50.0)	13 (25.5)	.007
**PCI status (no/yes)^b^**	31 (91.2)/3 (8.8)	9 (69.2)/4 (30.8)	
**Liver progression^a^, *n* (%)**	19 (27.9)	24 (47.1)	.032
**Lung progression^a^, *n* (%)**	30 (44.1)	22 (43.1)	.915
**Bone progression^a^, *n* (%)**	20 (29.4)	21 (41.2)	.181
**Lymph node progression^a^, *n* (%)**	23 (33.8)	19 (37.3)	.698

Abbreviation: PCI, prophylactic cranial irradiation.

^a^The progression sites were not available for 11 of the 79 patients in the 4-cycle group and 12 of the 63 patients in the 6-cycle group.

^b^Among patients who had intracranial progression, 8.8% had PCI in the 4-cycle group and 30.8% had PCI in the 6-cycle group.

**Figure 2:**
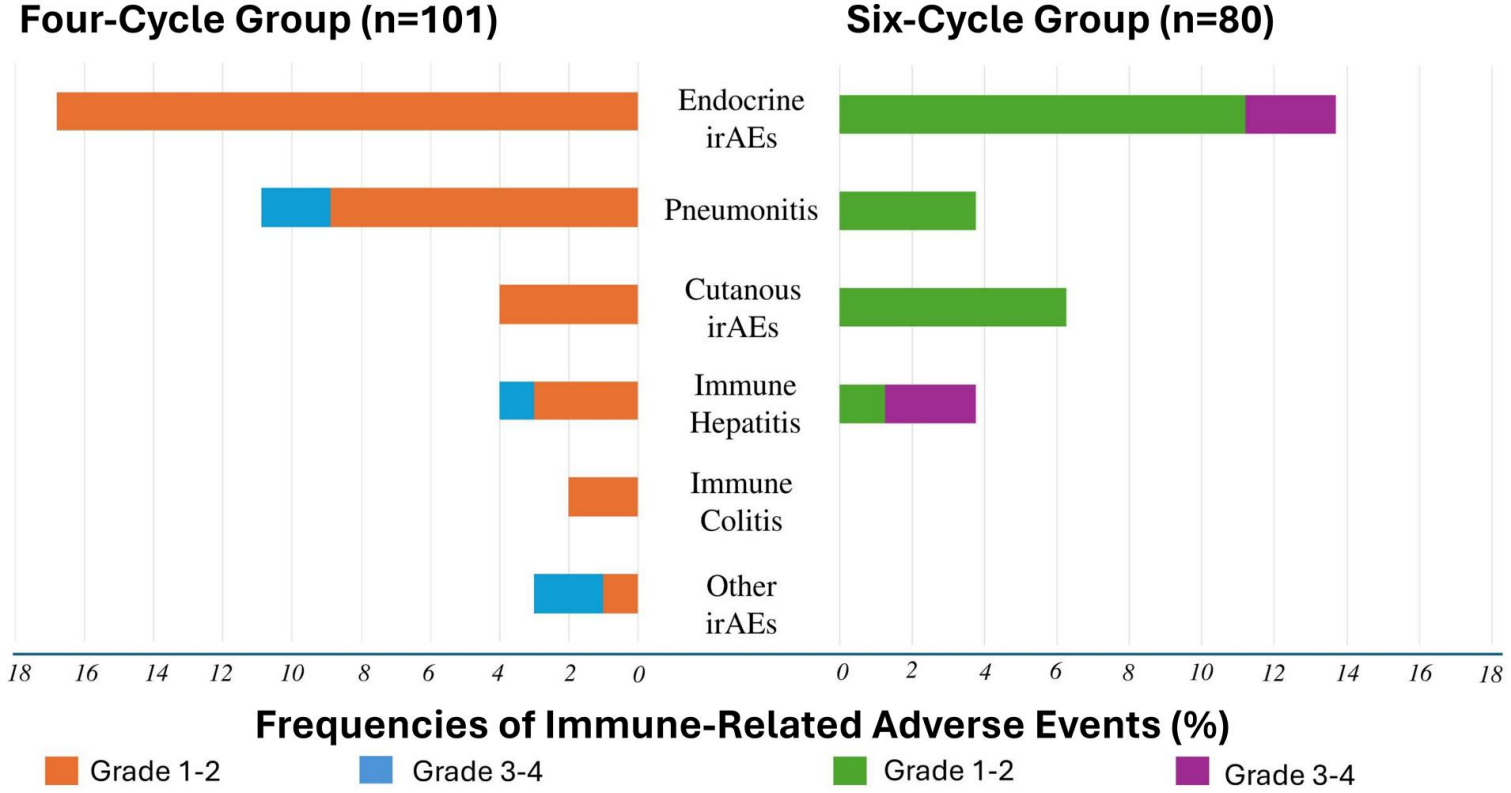
Immune-related adverse events according to number chemotherapy cycles. Any-grade immune-related adverse events (irAEs) were observed in 32.7% and 26.3% of patients in the 4- and 6-cycle groups, respectively (*P* = .348). Grade 3-4 irAEs occurred in 5.0% of patients in both groups. Endocrine toxicity was the most frequent irAE (15.8% vs 13.8%), followed by pneumonitis (10.9% vs 3.8%) in the 4- and 6-cycle groups, respectively. No grade 5 irAEs were observed.

Immune-related adverse events were observed in 33 (32.7%) and 21 (26.3%) patients in the 4-and 6-cycle groups, respectively (*P* = .348). The most frequent irAEs in both study groups were endocrine organ toxicities, followed by pneumonitis. Endocrinological irAE frequencies were 15.8% and 13.8% in the 4- and 6-cycle groups, respectively. Pneumonitis frequencies were 10.9% and 3.8%, respectively. Grade 3 or 4 irAEs were reported in 4 patients (5.0%) in the 4-cycle group, of which 2 had pneumonitis and one had hepatitis. Grade 3 or 4 irAEs in the 6-cycle group were 2 patients with endocrine toxicities and 2 patients with immune hepatitis, making 4 patients (5.0%) overall. There were no grade 5 irAEs in either of the study groups (Figure 2).

### Univariate and multivariate analyses for progression-free survival and overall survival

In the univariate analysis for PFS, liver metastasis (HR: 2.12, 95% CI: 1.44-3.13, *P* = .001), PCI (HR: 0.56, 95% CI: 0.36-0.85, *P* = .007), number of metastatic sites (HR: 1.65, 95% CI: 1.18-2.32, *P* = .004), and presence of irAEs (HR: 0.62, 95% CI: 0.43-0.89, *P* = .01) were statistically significant. The other factors included in the multivariate analysis were sex (HR: 0.66, 95% CI: 0.44-1.00, *P* = .051) and Charlson comorbidity index (HR: 1.26, 95% CI: 0.88-1.80, *P* = .201). According to the multivariate analysis for PFS, the number of Carbo/Etop plus atezolizumab cycles was not statistically significant (HR: 0.75, 95% CI: 0.54-1.06, *P* = .102), while the presence of liver metastasis (HR: 1.68, 95% CI: 1.19-2.39, *P* = .003), PCI (HR: 0.54, 95% CI: 0.35-0.83, *P* = .005), and irAEs (HR: 0.67, 95% CI: 0.46-0.96, *P* = .031) were independent significant factors affecting PFS (Table 3).

**Table 3:** Univariate and multivariate analyses of variables for progression-free survival.

*n* = 181	Univariate analysis HR (95% CI)	*P* value	Multivariate analysis HR (95% CI)	*P* value
**Age**		.346		
**<65 years**	Reference			
**≥65 years**	1.17 (0.83-1.65)			
**Sex**		.051		.117
**Female**	Reference		Reference	
**Male**	0.66 (0.44-1.00)		0.72 (0.47-1.09)	
**ECOG PS**		.542		
**0-1**	Reference			
**≥2**	1.13 (0.76-1.69)			
**Charlson Comorbidity Index**		.201		.573
**<9**	Reference		Reference	
**≥9**	1.26 (0.88-1.80)		1.11 (0.77-1.59)	
**Body mass index^a^ (kg/m^2^)**		.463		
**<25**	Reference			
**≥25**	0.88 (0.63-1.24)			
**Oncological emergency at presentation**		.332		
**No**	Reference			
**Yes**	1.32 (0.76-2.29)			
**Number of metastatic sites**		.004		.333
**<3**	Reference		Reference	
**≥3**	1.65 (1.18-2.32)		1.21 (0.82-1.78)	
**Brain metastasis**		.330		
**No**	Reference			
**Yes**	0.82 (0.54-1.23)			
**Liver metastasis**		.001		.003
**No**	Reference		Reference	
**Yes**	1.74 (1.24-2.41)		1.68 (1.19-2.38)	
**PCI**		.007		.005
**No**	Reference		Reference	
**Yes**	0.56 (0.36-0.85)		0.54 (0.35-0.83)	
**Thoracic radiotherapy**		.703		
**No**	Reference			
**Yes**	0.89 (0.49-1.61)			
**Carbo/etop-atezolizumab cycles**		.359	Reference	.102
**4 cycles**	Reference		0.75 (0.54-1.06)	
**6 cycles**	0.86 (0.61-1.19)			
**irAEs**		.01		.031
**No**	Reference		Reference	
**Yes**	0.62 (0.43-0.89)		0.67 (0.46-0.96)	

Abbreviations: Carbo/Etop, carboplatin/etoposide; ECOG PS, Eastern Cooperative Oncology Study Group performance status; HR, hazard ratio for progression; irAEs, immune-related adverse events; PCI, prophylactic cranial irradiation.

^a^Body mass index was not available for 2 patients.

The number of metastatic sites (HR: 1.81, 95% CI: 1.23-2.66, *P* = .003), and liver metastasis (HR: 2.12, 95% CI: 1.44-3.12, *P* < .001) were significant risk factors in the univariate analysis for OS. Age (HR: 1.38, 95% CI: 0.94-2.03, *P* = .100), ECOG PS (HR: 1.29, 95% CI: 0.82-2.04, *P* = .274), Charlson comorbidity index (HR: 1.28, 95% CI: 0.86-1.90, *P* = .234), presence of oncological emergency at presentation (HR: 1.57, 95% CI: 0.86-2.86, *P* = .144), and presence of irAEs (HR: 0.73, 95% CI: 0.48-1.11, *P* = .138) were included in the multivariate analysis. In the multivariate analysis, the number of carbo/etop plus atezolizumab cycles was not statistically significant (HR: 0.93, 95% CI: 0.63-1.38, *P* = .710). However, the presence of liver metastasis (HR: 2.31, 95% CI: 1.55-3.45, *P* < .001), PCI (HR: 0.57, 95% CI: 0.35-0.93, *P* = .024), and ECOG PS (HR: 1.63, 95% CI: 1.02-2.60, *P* = .041) were independent risk factors for OS (Table 4).

**Table 4:** Univariate and multivariate analyses of variables for overall survival.

*n* = 181	Univariate analysis HR (95% CI)	*P* value	Multivariate analysis HR (95% CI)	*P* value
**Age**		.100		.277
**<65 years**	Reference		Reference	
**≥65 years**	1.38 (0.94-2.03)		1.24 (0.84-1.84)	
**Sex**		.544		
**Female**	Reference			
**Male**	0.86 (0.52-1.41)			
**ECOG PS**		.274		.041
**0-1**	Reference		Reference	
**≥2**	1.29 (0.82-2.04)		1.63 (1.02-2.60)	
**Charlson Comorbidity Index**		.234		.764
**<9**	Reference		Reference	
**≥9**	1.28 (0.86-1.90)		1.07 (0.69-1.67)	
**Body mass index^a^ (kg/m^2^)**		.956		
**<25**	Reference			
**≥25**	0.99 (0.67-1.45)			
**Oncological emergency at presentation**		.144		.464
**No**	Reference		Reference	
**Yes**	1.57 (0.86-2.86)		1.27 (0.67-2.38)	
**Number of metastatic sites**		.003		.414
**<3**	Reference		Reference	
**≥3**	1.81 (1.23-2.66)		1.22 (0.76-1.94)	
**Brain metastasis**		.886		
**No**	Reference			
**Yes**	0.97 (0.61-1.53)			
**Liver metastasis**		<.001		<.001
**No**	Reference		Reference	
**Yes**	2.12 (1.44-3.12)		2.31 (1.55-3.45)	
**PCI**		.058		.024
**No**	Reference		Reference	
**Yes**	0.63 (0.40-1.02)		0.57 (0.35-0.93)	
**Thoracic radiotherapy**		.729		
**No**	Reference			
**Yes**	1.11 (0.61-2.03)			
**Carbo/etop-atezolizumab cycles**		.584		.710
**4 cycles**	Reference		Reference	
**6 cycles**	1.11 (0.76-1.63)		0.93 (0.63-1.38)	
**irAEs**		.138		.402
**No**	Reference		Reference	
**Yes**	0.73 (0.48-1.11)		0.83 (0.55-1.28)	

Abbreviations: Carbo/etop: carboplatin/etoposide; ECOG PS, Eastern Cooperative Oncology Study Group performance status; HR, hazard ratio for death; irAEs, immune-related adverse events; PCI, prophylactic cranial irradiation.

^a^Body mass index was not available for 2 patients.

## Discussion

We found that extending the induction carbo/etop regimen in combination with atezolizumab from the standard 4 to 6 cycles did not significantly improve either PFS or OS. In addition, the response rates were similar between the 4- and 6-cycle groups. The irAEs were also similar between the study groups and were generally low grade. The intracranial progression rate was significantly lower in the 6-cycle group. We demonstrated that the presence of liver metastasis was negative and that PCI was a positive prognostic factor for both PFS and OS. Patients with irAEs had significantly favorable PFS in both the univariate and multivariate analyses, whereas there was no significant effect on OS.

IMpower133 was the pivotal phase-3 study of carbo/etop plus atezolizumab. The frequency of brain metastasis was higher in our 4- and 6-cycle groups than in the atezolizumab arm of the IMpower133 trial (17.8% and 25.0% vs 8.5%). Moreover, IMpower133 reported that all patients had an ECOG PS of 0 or 1, whereas our cohort included patients with an ECOG PS ≥2 (21.8% and 17.2% in the 4- and 6-cycle groups, respectively). In the IMpower133 trial, the objective response rate (60.2%) in the atezolizumab arm was lower than that in our study groups, but the disease control rate (81.1%) was similar. The median PFS of the atezolizumab arm was 5.2 months and median OS was 12.3 months in the original study.^6^^,^^7^ Both the 4- and 6-cycle groups of our study had longer median PFS (6.3 and 8.1 months, respectively) and OS (16.6 and 13.6 months, respectively) than those in the IMpower133 trial. Despite having unfavorable baseline features in our study cohort, we observed longer survival outcomes in our study. The longer survival observed in our study may, in part, reflect increased chemotherapy exposure; however, this interpretation should be approached with caution, given the inherent selection bias of the retrospective design. Notably, our real-world cohort comprised patients who had already completed 4 to 6 cycles of chemotherapy, thereby excluding individuals with early disease progression or those unable to complete the planned treatment. In contrast, IMpower133 reported outcomes based on the full intention-to-treat population, capturing all randomized patients, irrespective of treatment completion. Although the proportion of chemotherapy discontinuation in IMpower133 was relatively low (2.5% for carboplatin and 4.0% for etoposide), and thus unlikely to fully account for survival differences, the reported dose intensities for carboplatin and etoposide were 92.3% and 89.4%, respectively. Collectively, these methodological differences underscore the need to interpret survival outcomes between retrospective real-world analyses and randomized clinical trials within the context of patient selection and treatment exposure. Moreover, irAEs were reported in 39.9% of patients in IMpower133, including 1.5% grade 5 toxicities.^6^ These toxicity proportions were higher than those in our study groups. In the phase-3b MAURIS study, 43 patients received 4 cycles and 89 patients received 5 or 6 cycles of induction carbo/etop plus atezolizumab regimen.^13^ For the 4-cycle and 5-6 cycle groups, the objective response rates were 72.1% and 84.3%, mPFS were 4.5 and 5.8 months and the mOS were 10.4 and 13.7 months, respectively (Table S1). Consistent with our results, they reported that prolongation of the induction phase of the carbo/etop regimen over 4 cycles did not significantly improve survival. Despite having similar response rates to those in our study, the survival outcomes were shorter than in our study groups. Moreover, the other common results of the MAURIS trial with our study findings were the favorable prognosis of patients who experienced irAEs and the poor prognostic effect of basal liver metastases. However, they did not include PCI as a prognostic variable in their analysis.^14^ In a study by Demir et al., 24 patients who received a median of 6 (6-12) cycles of induction carbo/etop and atezolizumab were analyzed. In their cohort, the median PFS was 9.5 months and the median OS was 30.1 months that associated the prolongation of the induction Carbo/Etop regimen with longer survival.^15^ Beyond atezolizumab, in a phase 3 b study (LUMINANCE), the patients received 4-6 cycles of platinum plus etoposide in combination with durvalumab.^16^^,^^17^ Despite not reporting a direct comparative survival analysis in accordance with induction regimen cycles, they demonstrated that mPFS was 6.3 months and mOS was 16.4 months in their study population, which was composed of 152 patients. The incidence of irAEs was 14.5%. Their immunotherapy-related toxicity ratio was notably different from our findings (32.7% in the 4-cycle group and 26.3% in the 6-cycle group). These variations in survival outcomes between different studies may be explained by molecular features beyond the differences in clinical and treatment characteristics. Tumor mutation burden was significantly associated with immunotherapy efficacy in patients with SCLC, despite the controversial role of PD-L1.^18^ SCLC has been classified into 4 distinct molecular subtypes; ASCL1, NEUROD1, POU2F, and YAP1. The YAP1 subset differs from the others in that it has a more immune-inflamed microenvironment and neoantigenicity.^19^ Nevertheless, our data did not include tumor mutation burden and PD-L1 values or molecular subset analyses of patients. Moreover, in a recent study, addition of lurbinectedin to maintenance atezolizumab improved survival outcomes.^20^

We demonstrated that liver metastasis had a negative association, whereas PCI had a positive association with both PFS and OS. Because PCI is delivered after induction and typically in patients without early progression, the observed association between PCI and improved outcomes may partly reflect confounding by indication, rather than a direct treatment effect of PCI. Thus, the PCI findings in this dataset are hypothesis-generating. Baseline brain metastasis was not an independent risk factor for PFS or OS. The number of metastatic sites had a significant p-value in univariate analyses for PFS and OS. Nevertheless, this significance was not valid in multivariate analyses. Explaining the negative association with prognosis in our study, immunotherapy efficacy was found to be lower in patients with liver metastasis for many cancer types.^21^ In a SEER database analysis, 24 507 patients with ES-SCLC were evaluated, and liver metastasis was found to be the most unfavorable prognostic factor, regardless of immunotherapy.^22^ Although statistical significance was not reached, it is noteworthy that the incidence of liver metastasis was numerically higher in the group receiving 6 cycles of treatment (48.8% vs 34.7%), which might be relevant for the interpretation of survival results of our study cohorts.

Brain metastasis was also associated with poor prognosis in patients with ES-SCLC.^23^ The rates of 1-year survival were found to be 18.0% for liver metastasis and 40.9% for brain metastasis.^24^ However, effective local treatment for brain metastases has been shown to improve survival outcomes.^25^ Considering the PCI application in extensive stage disease, there are phase-3 trials showing no survival benefit by PCI in ES-SCLC.^26^ A meta-analysis of PCI including 26 467 ES-SCLC cases, demonstrated that PCI was associated with improved OS. Nevertheless, the proportion of patients treated with immunotherapy was not reported in the meta-analysis. Another point of this meta-analysis was the insufficiency of radiological cranial screening. The patients who were radiologically proven to not have brain metastasis were found to decrease the positive prognostic effect of PCI, which is suggested to be a possible unintentional therapeutic effect of PCI, rather than a prophylactic effect.^27^ In this context, our findings should be interpreted cautiously. In our cohort, most patients with baseline brain metastases received cranial radiotherapy, regardless of the treatment group, which may have mitigated the survival differences attributable to PCI. Notably, intracranial progression was less frequent in the 6-cycle group, particularly among patients without baseline brain metastases. Nevertheless, consistent with the existing literature, no validated clinical or treatment-related factors have yet been clearly established to reliably predict intracranial progression in patients with ES-SCLC.^28^ Additionally, the comparable distribution of patients receiving PCI across the 2 study groups minimized the potential for PCI to act as a confounding factor in terms of intracranial progression. The immunotherapy era in SCLC has brought new challenging toxicities.^29^ We demonstrated that the presence of irAEs was significantly associated with increased PFS but not with OS. Recent studies have investigated the presence, onset time, grade, and type of irAEs as predictive markers of treatment efficacy. In patients receiving PD-1 and PD-L1 blockage, the presence of irAEs was associated with improved PFS and OS in the advanced stages of non-small-cell lung cancer, gastric cancer, urothelial carcinoma, and head and neck cancer.^30^ However, in a Japanese cohort of 40 patients with ES-SCLC, the presence of irAEs was not associated with survival outcomes study.^31^

The major limitation of our study is its retrospective design. As a result of the retrospective data collection, the detailed chemotherapy toxicity profile of the study population and the progression sites for 23 of 142 patients who had disease progression (16.2%) were missing, which may represent a limitation of our analysis. Another limitation of our study is that our data did not include PFS-2 analysis. Moreover, the risk of immortal time bias cannot be fully excluded, as treatment exposure was defined by the number of chemotherapy–immunotherapy cycles received. Although landmark analysis at predefined time points could mitigate this bias, the structure of the dataset and the limited sample size did not allow for a methodologically robust implementation. Additionally, brain imaging schedules were not standardized across centers. Patients receiving longer induction therapy may have undergone imaging at different intervals, potentially influencing the timing and detection of intracranial progression. Detailed data regarding chemotherapy dose reductions and relative dose intensity were not consistently available across participating centers; therefore, they could not be reliably analyzed. In addition, post-progression treatment selection was not available in many centers. However, being the first study in the literature that was directly designed to compare 4 and 6 cycles of induction of the carbo/etop and atezolizumab regimen is the strength of our study.

## Conclusion

In this multicenter, real-world ES-SCLC cohort, extending induction Carbo/Etop plus atezolizumab to 6 cycles did not improve PFS or OS. Lower intracranial progression with 6 cycles warrants cautious interpretation due to confounding factors and nonstandardized imaging. Prospective studies are needed to define the subgroups that may benefit from longer induction or PCI strategies.

## Supplementary Material

oyag092_Supplementary_Data

## Data Availability

The data underlying this article will be shared on reasonable request to the corresponding author.
