# Supplementary material for: Comparison of 6 cycles with 4 cycles of chemotherapy and atezolizumab in the first-line treatment of ES-SCLC: a retrospective multicenter analysis
Source: Oncologist. 2026 Mar 16;31(4):oyag092. doi: 10.1093/oncolo/oyag092 (PMC13049592; doi:10.1093/oncolo/oyag092)
Supplement: oyag092_Supplementary_Data [file oyag092_supplementary_data.docx]

| **Supplementary Table: Summary of the Results of the Current Study and the Previous Clinical Trials** | | | |
| --- | --- | --- | --- |
|  | **Treatment Regimen** | **Four Cycle Arm** | **Six Cycle Arm** |
|  |  | \| **PFS** \| **OS** \| \| --- \| --- \| | \| **PFS** \| **OS** \| \| --- \| --- \| |
| **Current Study** | A + Carbo/Etop | \| 6.3 \| 16.6 \| \| --- \| --- \| | \| 8.1 \| 13.6 \| \| --- \| --- \| |
| **IMpower133** **¶** | A + Carbo/Etop | \| 5.2 \| 12.3 \| \| --- \| --- \| | \|  \|  \| \| --- \| --- \| |
| **Mauris** | A + Carbo/Etop | \| 4.5 \| 10.4 \| \| --- \| --- \| | \| 5.8* \| 13.8* \| \| --- \| --- \| |
| **Caspian ¶** | D+ Platinum/Etop | \| 5.4 \| 12.9 \| \| --- \| --- \| | \|  \|  \| \| --- \| --- \| |
| ***:** In the MAURIS trial, patients who received five or six cycles of induction chemoimmunotherapy were analyzed together as the extended chemoimmunotherapy group  **¶:** IMpower133 and Caspian trials did not allow extended induciton regimen over four cycles  **A + Carbo/Etop;** Atezolizumab plus carboplatin and etoposide, **D + Platinum/Etop;** Durvalumab plus either cisplatin or carboplatin and etoposide, **OS;** Overall survival, **PFS;** Progression-free survival | | | |
